# Molecular characterization and evolution of the resident population of some alfalfa mosaic virus (AMV) isolates in Egypt

**DOI:** 10.1186/s12866-023-03003-8

**Published:** 2023-09-18

**Authors:** Hala A. Amin, H. A. Younes, Radwa M. Shafie, Mervat M. Fathallah

**Affiliations:** 1 Virus and Phytoplasma Research Department, Plant Pathology Research Institute, Agricultural Research Center (ARC), P.O. Box 12619, Giza, Egypt; 2 Agricultural Botany Department, Faculty of Agriculture, Alexandria University, Saba Basha, Alexandria, Egypt

**Keywords:** Alfalfa mosaic virus (AMV**)**, Coat protein gene (CP), Sequencing, Host antioxidant enzyme activity, And phylogenetic analysis

## Abstract

**Background:**

Alfalfa mosaic virus (AMV) is an important virus affecting many vegetable crops in Egypt. In this study, virus isolates were collected from naturally infected potato, tomato, alfalfa and clover plants that showed suspected symptoms of AMV in different locations of Beheira and Alexandria governorates during the 2019–2020 growing season. The relative incidence of the virus ranged from 11–25% based on visual observations of symptoms and ELISA testing. A total of 41 samples were tested by ELISA using polyclonal antisera for AMV. Four AMV isolates collected from different host plants, named AM1 from potato, AM2 from tomato, AM3 from alfalfa and AM4 from alfalfa, were maintained on *Nicotiana glutinosa* plants for further characterization of AMV.

**Results:**

Electron micrographs of the purified viral preparation showed spheroidal particles with a diameter of 18 nm and three bacilliform particles with lengths of roughly 55, 68, and 110 nm and diameters identical to those of the spheroidal particles. The CP gene sequence comparisons of four AMV isolates (AM1, AM2, AM3 and AM4) showed the highest nucleotide identity of 99.7% with the Gomchi isolate from South Korea infecting Gomchi *(Ligularia fischeri*) plants. Phylogenetic analysis showed that the present isolates were grouped together into a distinct separate clade (GPI) along with the Gomchi isolate from South Korea. Similarly, the deduced amino acid sequence comparisons of Egyptian AMV isolates revealed that amino acids Q^29^, S^30^, T^34^, V^92^ and V^175^ were conserved among the Egyptian isolates in GPI.

**Conclusion:**

The present study found strong evolutionary evidence for the genetic diversity of AMV isolates by the identification of potential recombination events involving parents from GPI and GPII lineages. Additionally, the study found that Egyptian AMV isolates are genetically stable with low nucleotide diversity. Genetic analysis of the AMV population suggested that the AMV populations differ geographically, and AMV CP gene is under mild purifying selection. Furthermore, the study proposed that the Egyptian AMV population had common evolutionary ancestors with the Asian AMV population. Antioxidant enzymes activity was assessed on *N. glutinosa* plants in response to infection with each AMV isolate studied, and the results revealed that the enzyme activity varied.

**Supplementary Information:**

The online version contains supplementary material available at 10.1186/s12866-023-03003-8.

## Background

Alfalfa mosaic virus (AMV) is one of the most significant viruses that infect many vegetable crops worldwide. Alfalfa mosaic virus is a species of the genus *Alfamovirus* belonging to the family *Bromoviridae* [1, 2]*.* AMV is a multipartite virus containing four particles measuring 18 nm in diameter (three bacilliform and one spheroidal). The length of viral bacilliform particles ranges from 30 to 57 nm, and they have hemispherical ends with pentagonal symmetry and a cylindrical portion with various hexamer expansions. The symmetry of icosahedral particles ranges from spherical to elongated [1, 3]. AMV contains tripartite single-stranded RNA genomes (RNA1, RNA2, and RNA3). The three genomic RNAs and subgenomic RNA4 (which is generated by negative-sense strand transcription of RNA3) are separately encapsidated into bacilliform particles [3]. RNA1 and RNA 2 consist of a single open reading frame (ORF) that encodes viral P1 and P2 replicase subunits, respectively. The movement protein and the coat protein (CP) are encoded by two ORFs in RNA3. The presence of RNA4 or AMV-CP translation products is important for RNA replication, viral RNA packaging, aphid transmission, and systemic infection [3, 4]. The AMV host range includes more than 600 species from 70 families. Although most of them are herbaceous plants, such as pepper (*Capsicum annum* L.), celery (*Apium graveolens* L.), bean (*Phaseolus vulgaris* L.), pea (*Pisum sativum* L.), lettuce (*Lactuca sativa* L.), tomato (*Solanum lycopersicum* L.), potato (*Solanum tuberosum* L.), alfalfa (*Medicago sativa* L.) and eggplant (*Solanum melongena* L.), AMV can also infect woody plant species (e.g., *Chinese wisteria* (*Wisteria sinensis*) tree) [5–8]. Recently, in Italy, AMV was first detected in the chayote plant (*Sechium edule*), which is a climbing cucurbit [9]. Natural AMV infection is less common in tomato plants than in pepper plants; however, the virus has the potential to cause significant economic losses when infecting tomato plants [10]. In Egypt, AMV infection has been reported in potatoes, alfalfa, and pepper [11–14]. It was isolated from naturally infected potato plants in the Nubaria region of Beheira governorate, which showed bright yellow (calico) symptoms [15]. AMV symptoms in alfalfa (*Medicago sativa* L. complex) can vary depending on the host genotype. While AMV infection can be asymptomatic in some cases, malformations, mosaic, and mottling symptoms are prevalent [12, 16]. The wide diversity of AMV host plants confirms the great ability of the virus to overwhelm the natural defensive responses of plants [17, 18].

Plants possess a variety of active defense mechanisms that can be effectively expressed in reaction to biotic stresses, such as pathogens. During host/pathogen interactions, peroxidase and chitinase are two types of pathogenesis-related proteins (PRs) that play key roles as anti-pathogenic factors [19]. H_2_O_2_ accumulation can act against the pathogen in two ways: it can kill the pathogen directly, or it can prevent the pathogen from entering the host plant [20]. Furthermore, it helps to strengthen cell walls by enhancing peroxidase reactions that stimulate intra- and intermolecular cross-links between structural components of the cell wall and lignin polymerization [21]**.** Plant polyphenol oxidases (PPOs), which catalyze the O_2_-dependent oxidation of phenolics to quinones, have been proposed as a component of complex plant defense mechanisms [22, 23]. In addition, antioxidant enzymes such as SOD, CAT, POX, and PAL play a distinctive role in reducing the impacts of oxidative stress induced by AMV infection [24].

In the *Bromoviridae* family, the CP gene mutations reverse the amino acid sequence of CP, thus altering the transmissibility of aphids and the formation of unusually long virus particles even if the mutation occurs in only one amino acid [1, 25–27]. AMV isolates were grouped into different clades, which has been well documented by different authors worldwide [16, 28–32]. The topology trees for the Italian and French AMV strains grouped them into two monophyletic groups (subgroup I and II) and appear to propose the influence of geographic distinctiveness on the evolutionary dynamics of these AMV strains that is not associated with differences in host symptoms [17]. The nucleotide similarity of eight Canadian AMV isolates from potato was established by sequence analysis of their CP gene. Sequence comparisons and phylogenetic analysis clustered these AMV isolates into a single cluster [30].

The determinants of genetic and viral population structure are associated with evolutionary forces such as mutation, genetic exchange, migration, selection, and genetic drift. They can influence vector transmission and the relative effect of environmental factors related to plant hosts and virus epidemiology [33]. Little is known about the characterization of AMV isolates in Egypt. Knowledge of the diversity and genetic structure of AMV populations, especially Egyptian isolates, is scarce, and only a few studies provide information on variation among isolates based on nucleotide and amino acid sequences [13, 24, 34–36]. For example, the AMV isolate from Assiut (Assuit-AMV) is similar to Egyptian AMV isolates from Wadi El Natrun and Menoufia governorates, where the degree of variation was only 4 and 5.6%, respectively. Additionally, Assuit-AMV isolates and global AMV isolates tend to cluster into two major groups, and this trend may indicate the existence of two distinct strains of AMV and two evolutionary pathways for AMV isolates [34]. On the other hand, phylogenetic analysis of the Egyptian AMV isolate (FER-AM1) suggested that it is a unique strain of AMV and belongs to a new subgroup IIC [35]. The CP phylogenetic analysis showed that Egyptian AMV-eggplant-EG isolates from Kafr El-Sheikh governorate are closely clustered with Egyptian isolates available in the database with bootstrap values of 88% and 92%, respectively [24]. Similarly, CP gene analysis of the AMV-Basil isolate collected from Beni Suif governorate, Egypt, showed a high degree of diversity (3.6-8.7%) among twenty AMV isolates from different parts of the world [36]. Therefore, a larger number of AMV sequences is required to give a clear idea about variations between AMV isolates. Information on virus variability can be valuable in understanding the evolution of the resident population and defining useful criteria for selecting species cultivars for AMV resistance [17].

In this study, we attempted to molecularly and evolutionarily evaluate the resident population of some AMV isolates in Egypt based on SSCP analysis, followed by nucleotide sequence, recombination and genetic diversity analysis. In addition, we determined the enzymatic activity of infected plants against present AMV isolates under greenhouse conditions.

## Results

### Detection of AMV

**Fig. 1:**
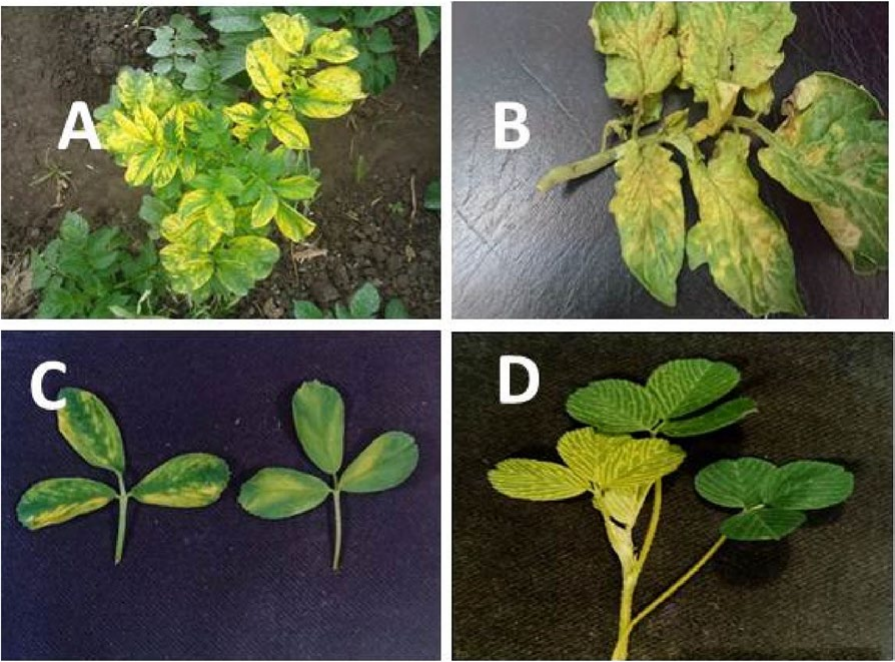
Symptoms of natural infection of AMV on **A** potato, **B** tomato, **C** alfalfa and **D** clover

During sample collection, various types of symptoms expressed by the plants, including calico, bright yellow, and mosaic with slightly curved leaf margins, were observed on naturally infected potato, tomato, alfalfa, and clover plants (Fig. 1). The relative disease incidence of AMV in tomato, potato, alfalfa, and clover fields varied among crops at a percentage rate of 11–25%. The number of plants collected from each crop and the number of natural infections with AMV that were tested by phenotypical symptoms of the virus and ELISA tests in three locations during the 2019–2020 season are shown in Table 1.

**Table 1:** Number of natural infections with AMV tested by ELISA in three locations during the 2019–2020 season

Infected hosts	Location	No. of samples infection
		**Total samples**
Potato	(Alexandria, Nubaria)	2/9
Tomato	(Alexandria, Abis)	2/11
Alfalfa	(Alexandria, Nubaria)	3/12
Clover	(Beheira, Wadi Natroun)	1/9

### Indirect ELISA

Serological detection using indirect ELISA in leaf samples from the tested plants detected AMV infection in symptomatic plants collected from different types of host plants (potato, tomato, alfalfa, clover). The results of the representative samples are presented in Table 2. Four samples with severe AMV mosaic symptoms (Table 2: No. 7 in potato, 2 in tomato, No. 4 in alfalfa, and No. 2 in clover, the samples are identified in bold text with an asterisk next to their ELISA values) showed the highest ELISA positive values (0.672, 0.705, 0.656 and 0.614 OD, respectively) ​​at a 405 nm wavelength (Table 2). These samples were used as a source of the virus isolates in the present study and given the names AM1, AM2, AM3 and AM4 (collected from potato, tomato, alfalfa and clover host plants, respectively). AMV isolates were mechanically transmitted from naturally infected plants to healthy *N. glutinosa* seedlings, which were used as a source of the virus isolates for subsequent studies in a greenhouse.

**Table 2:** Absorbance values of collected plants using indirect ELISA at 405 nm wavelength

No. of tested samples	Indirect ELISA absorbance values (A 405 nm)			
	**Potato**	**Tomato**	**Alfalfa**	**Clover**
1	0.227	0.235	**0.581**	0.302
**2**	0.339	**0.705**^**a**^	0.278	**0.614**^**a**^
3	0.327	0.262	0.352	0.276
**4**	**0.596**	0.381	**0.656**^**a**^	0.282
5	0.312	0.375	0.284	0.301
6	0.302	0.323	0.295	0.254
**7**	**0.672**^**a**^	0.384	0.240	0.332
8	0.320	**0.664**	0.271	0.346
9	0.272	0.320	o.396	0.295
10	….	0.276	0.281	……
11	……	0.376	0.371	……
12	……	……	**0.609**	……
H	0.264	0.276	0.284	0.281

The experiment was repeated twice, and ELISA absorbance values at 405 nm are the average of two replicates each. *H=* healthy. Bold = Positive reaction for each host plant. ^a^ = the highest ELISA-positive samples from each host used for further characterization in this study. – No samples collected

### Reaction of diagnostic hosts

**Fig. 2:**
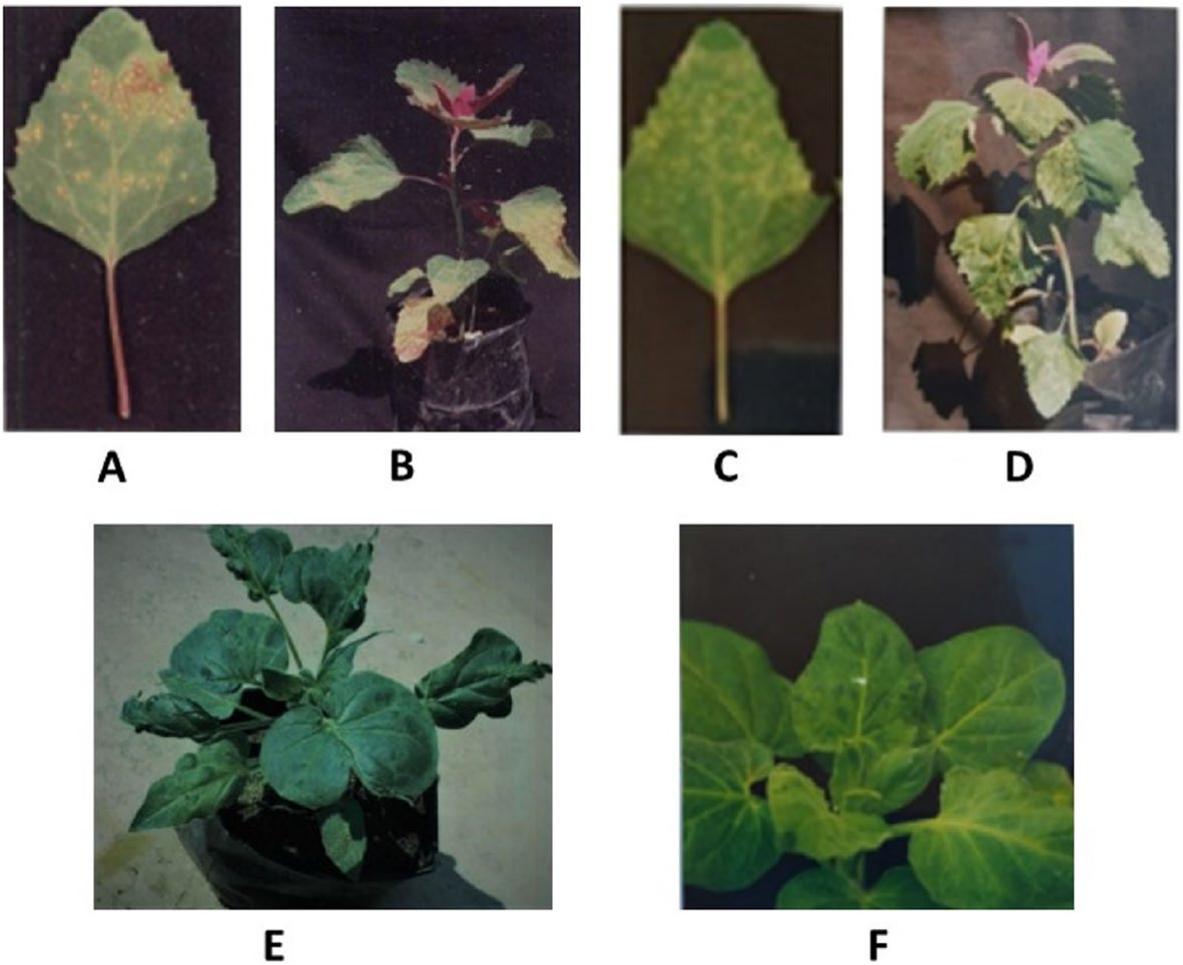
**A** Necrotic local lesions developed on *C. amaranticolor* leaves 7 days after inoculation with AM2. **B** Systemic symptoms on* C. amaranticolor* developed 25 days after inoculation with AM2. **C** Chlorotic local lesions developed on *C. amaranticolor* leaves 7 days after inoculation with AM1, AM3 and AM4. **D** Systemic symptoms on *C. amaranticolor* developed 25 days after inoculation with AM1, AM3 and AM4. **E** Reaction of *N. glutinosa* to infection with AM2, mottling on leaves, 15 days following leaf deformation, 35 days after inoculation. **F*** N. glutinosa* with infection by AM1, AM3, and AM4, mosaic symptoms developed 15 days after inoculation, followed by stunting

AMV collected from the field samples was successfully transmitted to different diagnostic hosts, such as *Chenopodium amaranticolor* and *N. glutinosa*. The plants inoculated with the virus in the four AMV isolates showed typical AMV symptoms. Isolate AM2 (from tomato) caused necrotic lesions 5–7 days post inoculation (dpi), followed by systemic symptoms at 20–25 dpi. However, isolates AM1, AM3, and AM4 (previously collected from potato, alfalfa, and clover, respectively) caused chlorotic local lesions at 5–7 dpi, followed by leaf deformation at 20–25 dpi on *C. amaranticolor.* (Fig. 2). *N. glutinosa* showed mottling symptoms at 15 dpi, followed by leaf deformation at 35 dpi with AM2, while AM1, AM3, and AM4 caused mosaic symptoms at 20 dpi, followed by stunting (Fig. 2).

### Enzymatic activity of plants infected with AMV

The activity of the antioxidant enzymes (SOD, CAT, APX and PPO) and H_2_O_2_ generation were assessed to evaluate the host reaction response to infection with the AMV isolates here studied (AM1, AM2, AM3, and AM4). Each AMV isolated from naturally infected plants was used to mechanically infect healthy *N. glutinosa* seedling plants in the greenhouse. The activity of antioxidant enzymes in response to infection with each AMV isolate on *N. glutinosa* plants differed, with a significant decrease in CAT (0.352 – 0.384 nKatl mg^−1^) and SOD (0.537–0.563 nKatl mg-1) activities in infected plants compared to healthy plants (0.553 and 0.719 nKatl mg^−1^ for CAT and SOD, respectively). In contrast, the activities of APX and PPO, and the generation of H_2_O_2_, were higher in infected plants relative to healthy control plants and the increase was significant after 4 days of infection as shown in Table 3.

**Table 3:** Enzymatic activity of plants infected with AMV

Treatment	SOD activity	CAT activity	APX activity	PPO activity	H_2_O_2_ content mmol/gFW
	**nkatl/mg protein**			**U/mL**	
**Control**	0.719^a^**	0.553^a^	0.162^b^	0.629^c^	4.584^b^
**AM1**	0.563^b^	0.368^b^	0.185^a^	1.663^a^	10.169^a^
**AM2**	0.537^b^	0.372^b^	0.188^a^	1.565^bc^	10.183^a^
**AM3**	0.551^b^	0.384^b^	0.197^a^	1.779^a^	10.176^a^
**AM4**	0.546^b^	0.352^b^	0.183^a^	1.775^a^	10.193^a^
**LSD**	0.29	0.05	0.03	0.10	0.03

Data are the average of four replicates. ** Means followed by the same letter are not significantly different at *P* ≥ .05 levels

Different lowercase letters (a, b and c) within the same column indicate significant differences between AMV isolates

### RT‒PCR detection

**Fig. 3:**
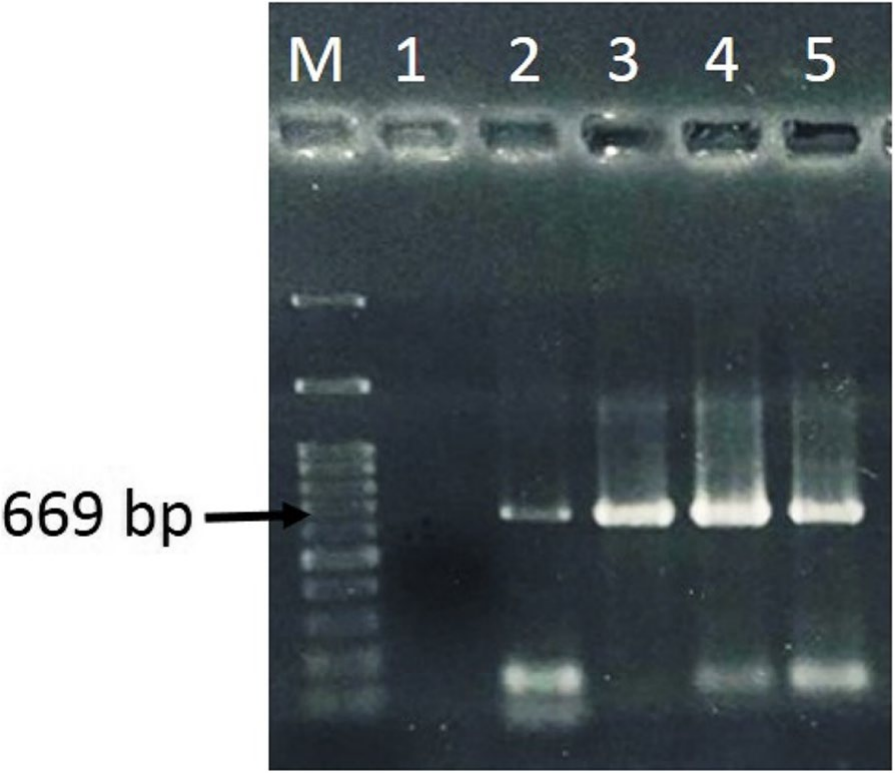
One percent agarose gel electrophoresis analysis of AMV CP gene amplicons obtained by RT‒PCR from mechanically inoculated *N. glutinosa* leaves. M: 100 bp DNA Ladder (GeneDireX, Inc.); lane 1: uninfected healthy *N. glutinosa* plant; lane 2: isolate AM1; lane 3: isolate AM2; lane 4: isolate AM3; and lane 5: isolate AM4. 'Full-length gel is presented in Additional file 1', Fig. 3

AMV isolates AM1-potato, AM2-tomato, AM3-alfalfa, and AM4-clover obtained from different locations in Egypt (Alexandria and Beheira governorates) and maintained in *N. glutinosa* plants were selected for total RNA extraction and further molecular analysis. The CP gene was amplified with the AMVF2/AMVR2 primer, yielding an expected amplicon size of 669 bp. No amplification product was observed from *N. glutinosa* uninoculated plants (Fig. 3).

### SSCP analysis

**Fig. 4:**
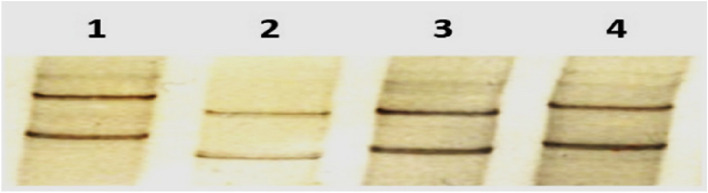
SSCP patterns of RT‒PCR products of all tested AMV isolates; lane 1: AM1 isolate; lane 2: AM2 isolate; lane 3: AM3 isolate, and lane 4: AM4 isolate. 'Full-length blot is presented in Additional file 1, Fig. 4

The SSCP pattern of the RT‒PCR products of the four AMV isolates under study was analysed. Each distinct SSCP pattern was then considered a haplotype. SSCP analysis showed haplotype matching for the AM1, AM3 and AM4 isolates, while the AM2 isolate showed a different SSCP pattern (Fig. 4). The SSCP patterns of the AM1, AM3, and AM4 isolates showed multiple DNA bands with two dense and predominant bands. On the other hand, the SSCP pattern for the AM2 isolate resulted in only two dense bands (Fig. 4). The purified PCR products of AM1, AM2, AM3 and AM4 isolates showing identical and different SSCP patterns were cloned and sequenced in both directions.

### Nucleotide sequence and phylogenetic analysis

The RT‒PCR amplicons of the 669 bp CP gene of the four selected AMV isolates AM1 (potato), AM2 (tomato), AM3 (alfalfa), and AM4 (clover) were cloned and sequenced. The sequence analysis showed 98.2 to 99.5% sequence identity at the nucleotide (nt) level within the present AMV isolates, as shown in supplementary (S1), and 97.3 to 99.1% identity at the deduced amino acid level (see Additional file 2). Nucleotide sequence comparisons of the present four AMV isolates along with 38 AMV isolates available in GenBank from 13 different countries revealed nt identities ranging from 89.6% to 99.7% (Table 4). Isolate AM3 showed the highest nucleotide identity (99.7%) with the Gomchi isolate from South Korea with accession No. LC219343.1 from Gomchi (*Ligularia fischeri*) host, while isolate AM2 showed the lowest identity (89.6%) with isolate Wadi Al-Dawasir from Saudi Arabia (KC434084—alfalfa host) (Table 4) when compared to foreign isolates.

**Table 4:** Accession numbers, isolate names, origins and identity matrix of AMV isolates used in this study

No	Isolate name	Accession No	Country	Host plant	Identity matrix (%)			
					**AM1**	**AM2**	**AM3**	**AM4**
1	Shark	LN846978	Egypt, Alexandria	tomato	99.5	98.2	98.8	99.1
2	M123	MK673272	Egypt, Alexandria	potato	99.2	98.1	98.7	99.0
3	EM143	MN335927	Egypt	potato	98.6	97.9	99.4	98.4
4	EM99	MN335926	Egypt	potato	97.9	97.0	98.4	97.9
5	AMV-EG	MW428250	Egypt, Kafr El-Sheikh	eggplant	96.5	96.1	96.4	96.4
6	AMV77	MN548391	Egypt, Alexandria	potato	95.5	95.5	95.1	95.3
7	AMV-Basil	MH625710	Egypt, Beni Suef,	basil	90.8	90.1	90.8	90.7
8	M3	HG315522	Egypt, Beheira, Itay	potato	98.5	97.1	98.0	98.5
9	Menoufia	HQ288892	Egypt, Menoufia	potato	93.5	93.4	93.8	93.4
10	Assiut	KT284729	Egypt, Assiut	potato	99.7	99.0	99.7	99.3
11	FER-AM1	KY176007	Egypt	potato	99.5	98.4	98.9	99.5
12	E3	MW846083	Egypt	beet	95.9	95.4	95.7	95.7
13	Lye 80	AJ130703	France	tomato	91.4	91.3	91.7	91.4
14	Caa 1	AJ130707		pepper	91.9	91.3	91.9	91.7
15	Dac 16	AJ130708		carrot	91.6	91.0	91.6	91.4
16	126A	AJ130704	Italy	parsley	92.4	92.4	92.7	92.2
17	195 AN	AJ130705		tomato	92.7	92.5	93.0	92.5
18	F430	AJ130706		bean	93.7	93.5	94.0	93.5
19	Gomchi	LC219343	South Korea	gomchi	99.2	98.2	99.7	99.1
20	KR1	AF294432		potato	92.9	92.5	92.9	92.8
21	Brazil	FJ858265	Brazil	alfalfa	92.5	93.3	92.5	92.3
22	AMV-Mexico	MG813772	Mexico	pepper	93.5	92.6	93.5	93.4
23	Manfredi	KC881010	Argentina	alfalfa	92.8	92.6	92.8	92.6
24	AMV 425 M	K02703.1	USA, Madison	clover	92.2	92.0	92.2	92.0
25	AMV 425 L	L00162.1	USA, Leiden	clover	92.2	91.7	92.2	92.0
26	Fa.Fa.A	KX535481	Iran	alfalfa	94.1	93.5	94.1	93.9
27	Ke.Ke.Pe	KX535513		pepper	94.2	93.9	94.2	94.1
28	Ke.Ji.Po	KX535512		potato	93.9	93.9	93.9	93.8
29	NZ2	U12510.1	New-Zealand	alfalfa	92.6	92.5	92.6	92.5
30	NZ34	AF215664			93.8	93.4	94.1	93.7
31	Wadi-aldawasir	KC434084	Saudi Arabia	alfalfa	89.8	89.6	89.8	89.8
32	AMV-TA1	KJ847776		alfalfa	92.6	92.6	92.9	92.6
33	VRU	AF015716.1	England	Garden lupine	93.5	93.2	93.4	93.4
34	strain S	X00819.1		alfalfa	93.2	92.8	93.2	93.1
35	258–11	KF147805	Serbia	tomato	94.3	94.0	94.3	94.2
36	95–08	FJ527748		Alfalfa,	93.7	93.2	93.7	93.5
37	196–08	FJ527749		tobacco	94.3	93.9	94.3	94.2
38	WC3(cp)	JN209847.1	Australia	clover	93.7	93.2	93.7	93.5

**Fig. 5:**
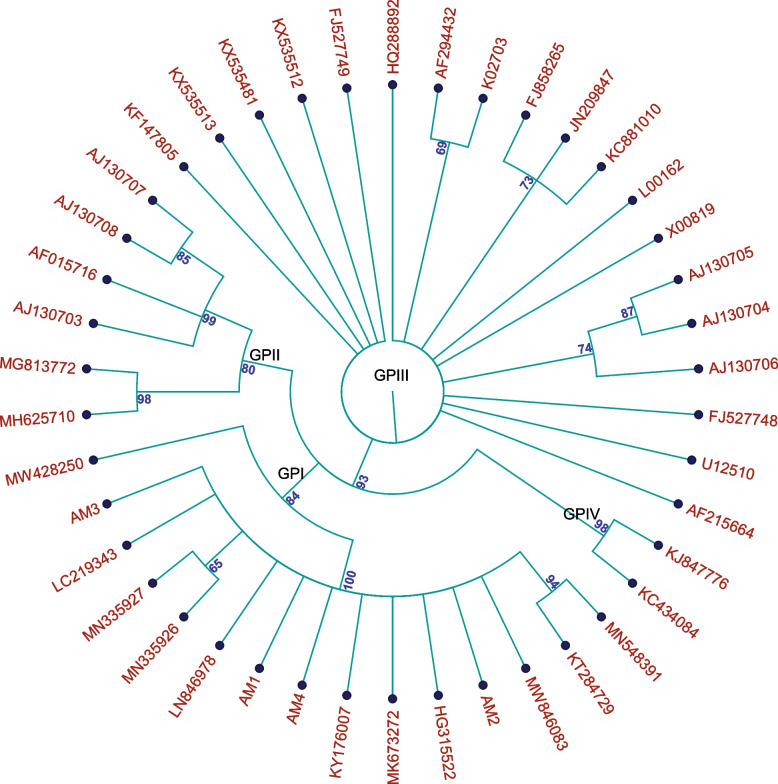
Rooted circular cladogram showing the phylogenetic tree of the AMV CP gene sequences of Egyptian isolates, including the current isolates (AM1, AM2, AM3 and AM4) and GenBank worldwide reference isolates. The neighbor-joining (NJ) phylogenetic tree was constructed using QIAGEN CLC Genomics Workbench software at 1000 bootstrap repetitions

Different levels of variation are observed in the CP gene sequences when compared with previously reported AMV isolates from GenBank. The Egyptian isolates AM1 (potato), AM2 (tomato), AM3 (alfalfa), and AM4 (clover) detected in this study shared the highest nucleotide identity (99.7%–95.1%) with other isolates from Egypt Assiut, FER-AM1, Shark, M123, M3, AMV77, E3, and AMV-EG reported previously from potato, tomato, beet, and eggplant hosts, respectively, as shown in Table 4 and supplementary (S1). In contrast, they shared low nucleotide sequence identities (ranging from 90.0% to 93.8%) with AMVs from Beni Suef (MH625710-basil) and Menoufia (HQ288892-potato) isolates (supplementary S1). The viral CP-deduced amino acid sequence comparison for the Egyptian AMV isolates revealed identities almost similar to those of nt sequences (see Additional file 2). The comprehensive topology of the circular cladogram of the neighbor-joining (NJ) phylogenetic tree of the nucleotide sequences of the coat protein gene of AMV (Fig. 5) revealed four distinct monophyletic groups that were classified as GPI, GPII, GP III, and GP IV. Almost all Egyptian isolates, such as FER-AM1 (KY176007-potato), Shark (LN846978-tomato), M123 (MK673272-potato), M3 (HG315522-potato), Assiut (KT284729-potato), and the present isolates (AM1, AM2, AM3, and AM4), were grouped together into a distinct separate clade (GPI) along with the Gomchi isolate from South Korea. Group GPII included isolates from France, Egypt, and Mexico, but the Egyptian AMV-Basil isolate and the AMV-Mexico isolate were grouped together into a separate clade as a distinct subgroup, as illustrated in Fig. 5. On the other hand, Group III (GPIII) included global AMV isolates retrieved from the GenBank database and isolated from Italy, Iran, the United States, New Zealand, Australia, Brazil, Serbia, and England, in addition to the Menoufia isolate from Egypt, as shown in Fig. 5. Group IV (GPIV) included Saudi Arabian isolates, as shown in Fig. 5. The names, accession numbers, and geographic origins of the AMV isolates are given in Table 4. From our phylogenetic tree topology, we noticed that AMV strains from Italy (AJ130704, AJ130705, and AJ130706), France (AJ130703, AJ130707, and AJ130708), Iran (KX535481, KX535513, and KX535512), and AMV strains (FJ858265, KC881010, and JN209847.1) from Brazil, Argentina, and Australia were separately grouped into subgroups. The nucleotide sequences of the current Egyptian AMV isolates studied in this investigation have been deposited in GenBank under the following accession numbers: OM105666 to OM105669.

The alignment of the deduced amino acid sequences revealed differences in the CP amino acids between the AMV strains of GP I and the AMV strains of the other groups at positions 29, 30, 34, 36, 66, 92, 93, 117, 132, 134, 136, and 175, as shown in Table 5. Group (GPI) showed amino acid substitutions as follows: glutamine (Q^29^) instead of Leucine (L), proline (P) replaced with serine (S^30^), threonine (T^34^) amino acid instead of alanine (A), Valine (V^92^) instead of Isoleucine (I), and Glutamic acid (E^117^) instead of Aspartic acid (D). In addition, glutamine (Q) in GPIII and Leucine (L) in GPII and GPIV were replaced with Valine (V^175^) in GPI, as indicated in Table 5. T^34^, E^117^, and V^175^ were marked for GPI only. While amino acids Q^29^ and V^92^ of GPI were characterized and specialized for all Egyptian isolates, they were also found in the Egyptian AMV-Basil isolate, which was clustered in group GP II. Comparative amino acid sequence analysis revealed two amino acid residues, phenylalanine (F^66^) and tyrosine (Y^93^), conserved among AMV isolates of GPI and GPIII. Based on phylogenetic analysis and comparisons of amino acid sequences of GPI AMV strains, including the present Egyptian isolates, they appear to be more closely related to GPIII, as shown in Fig. 5 and Table 5.

**Table 5:** Deduced amino acid differences of AMV isolates, including the present isolates

**Groups**	**Isolates name**	**Amino acids position**											
		**29**	**30**	**34**	**36**	**66**	**92**	**93**	**117**	**132**	**134**	**136**	**175**
**GP I**	AM1	**Q**	S	**T**	N	F	**V**	Y	**E**	Q	A	R	**V**
	AM2	**Q**	S	**T**	N	F	**V**	Y	**E**	Q	A	R	**V**
	AM3	**Q**	S	**T**	N	F	**V**	Y	**E**	Q	A	R	**V**
	AM4	**Q**	S	**T**	N	F	**V**	Y	**E**	Q	A	R	**V**
	Gomchi	**Q**	S	**T**	N	F	**V**	Y	**E**	Q	A	R	**V**
	Shark	**Q**	S	**T**	N	F	**V**	Y	**E**	Q	A	R	**V**
	M3	**Q**	S	**T**	N	F	**V**	Y	**E**	Q	A	R	**V**
	M123	L	P	**T**	N	F	**V**	Y	**E**	Q	A	R	**V**
	EM99	**Q**	S	**T**	N	F	**V**	Y	**E**	Q	A	R	**V**
	EM143	**Q**	S	**T**	N	F	**V**	Y	**E**	Q	A	R	**V**
	AMV-EG	**Q**	P	**T**	K	F	**V**	Y	**E**	Q	A	H	**V**
	AMV77	**Q**	S	**T**	K	F	**V**	Y	**E**	Q	A	R	**V**
**GPII**	Lye_80	L	P	A	K	S	I	F	D	H	A	H	L
	Caa_1	L	P	A	K	S	I	F	D	H	I	H	L
	Dac 16	L	P	A	K	S	I	F	D	H	I	H	L
	AMV-Mexico	L	P	A	K	S	V	F	D	Q	A	H	L
	AMV-Basil	**Q**	P	A	K	S	**V**	F	D	**Q**	A	H	L
	VRU	L	P	A	K	S	I	F	D	H	A	H	L
**GPIII**	126A	L	P	A	K	F	I	Y	D	Q	V	H	Q
	195_AN	L	P	A	K	F	I	Y	D	Q	V	H	Q
	F430	L	P	A	K	F	I	Y	D	Q	V	H	Q
	Brazil	L	P	A	K	F	I	Y	D	Q	V	H	Q
	AMV 425 L	L	P	A	K	F	I	Y	D	H	A	H	Q
	Fa.Fa.A	L	P	A	K	F	I	Y	D	H	A	H	Q
	Ke.Ji.PO	L	P	A	K	F	I	Y	D	H	A	H	Q
	NZ34	L	P	A	K	F	I	Y	D	H	A	H	Q
	WC3(cp)	L	P	A	V	F	I	Y	D	Q	V	H	Q
	95–08	L	P	A	K	F	I	Y	D	Q	V	H	Q
	strain S	L	P	A	K	F	I	Y	D	H	A	H	Q
	KR1	L	P	A	K	F	I	Y	D	Q	A	H	Q
**GPIV**	Wadi- aldawasir	L	P	A	K	S	I	Y	D	H	D	H	L
	AMV-TA1	L	P	A	K	S	I	Y	D	H	D	H	L

Amino acids characterizing group (GPI) and Egyptian isolates are written in bold text

### Reassortment/recombination analysis

The entire AMV CP gene sequence alignment was used to detect the recombination events in AMV isolates using the RDP4 program. The present AMV isolates (AM1-potato, AM2-tomato, AM3-alfalfa, and AM4-clover) showed no evidence of recombination when compared to those of 42 AMV strains retrieved from GenBank. On the other hand, our analysis found four putative reassortment/recombination events among 12 Egyptian AMV isolates (Old set retrieved from GenBank), as indicated in Table 6. The RDP4 software identified the isolates AMV77, M3, AMV-Basil, and Menoufia from Egypt (accession No. MN548391, HG315522, MH625710, and HQ288892, respectively) as potential recombinants for the CP gene (Table 6).

**Table 6:** Recombination events detected in alfalfa mosaic virus isolates

Recomb	Major parent	Minor parent	% similar	Av. *P*- value	Breaking^a^ points	Detection methods^b^						
						**R**	**G**	**B**	**M**	**C**	**S**	**T**
AMV77 (MN548391)	VRU (AF015716)	M3 (HG315522)	92.5%	1.145 × 10^–04^- 9.374 × 10^–05^	324—396	-	-	+	+	-	+	+
	EM99 (MN335926)	Dac 16 AJ130708	99.1%	1.227X10^−2^- 9.536X10^−5^	324—396	-	-	+	+	-	+	+
M3 (HG315522)	EM99 (MN335926)	AMV-Mexico (MG813772)	99.2%	7.316 × 10^–06^- 1.26 × 10^–02^	522—586	-	-	-	+	-	+	+
AMV-Basil (MH625710)	EM99 (MN335926)	AMV77 (MN548391)	93.3%	1.397X10^−2^—5.555X10^−3^	577—471	+	-	+	+	+	+	+
Menoufia (HQ288892)	EM99 (MN335926)	AMV77 (MN548391)	96.6%	1.397X10^−2^—5.555X10^−3^	523—464	+	-	+	+	+	-	-
AMV 425 L (L00162.1)	EM99 (MN335926)	AMV77 (MN548391)	98%	1.397X10^−2^—5.555X10^−3^	518—464	+	-	+	+	+	+	+

^a^Begin and end breakpoints positions in the recombinant sequence. ^b^RDP (R), GENECONV (G), BOOTSCAN (B), MAXICHI (M), CHIMAERA (C), SISCAN (S) and 3SEQ (T) recombination detection methods, % similar. = % of similarity, AV. *P*-value = Average of *P*- value

The isolates VRU (AF015716) from England (GPII lineage) and EM99 (MN335926) from Egypt (GPI lineage) were found to be major parents for recombination events of isolates M3 and AMV77, respectively, with percentages of similarity of 92.5% and 99.2%, respectively. Interestingly, the isolate EM99 from Egypt was detected as the major parent and the isolate AMV77 as the minor parent for the Egyptian recombinant isolates (AMV-Basil and Menoufia) and for the isolate AMV 425 L from USA, and the breaking point likely to start at 464 and end at 577 (Table 6). The obtained RDP algorithms, the breaking points, and the corresponding *P* values are reported in Table 6.

### Genetic diversity and evolution of the Egyptian AMV population

The genetic variation and polymorphism of the AMV isolates in the CP region were studied using DnaSP6. The analysis showed that all AMV isolates (*n* = 42) (Table 7) had a global selection pressure (dN/dS) of 0.6436 in the CP region. Haplotype (Hd) and nucleotide diversity (π) for AMV isolates were 0.995 and 0.08130, respectively. The haplotype (Hd) and nucleotide diversity (π) for Egyptian AMV population (*n* = 15) were 0.971 and 0.03787, respectively. Geographic and phylogenetic group populations were used to establish the genetic distinction of AMV populations. The haplotype (gene) diversity (Hd) for the phylogenetic groups GPI, GPII, GPIII, and GPIV within the population was 0.85714, 1.0000, 0.99415, and 0.99415, whereas the nucleotide diversity (π) for these four groups was 0.08976, 0.03957, 0.02402, and 0.03957, respectively.

**Table 7:** Genetic polymorphism estimated for CP region of AMV isolates

Variant group	h	Hd	π	dN	dS	dN/dS (ω)	Tajima’s D	Fu & Li’s D*	Fu & Li’s F*
AMV isolates (* n* = 42)	38	0.995 (± 0.007)	0.08130 (± 0.02550)	0.07397	0.11492	0.6436	-2.21986	-4.90837	-4.69093
Present AMV isolates (* n* = 4)	4	1.000 (± 0.177)	0.01855 (± 0.00667)	0.06461	0.10184	0.617	-0.59574 (n.s)	-0.59574 (n.s)	-0.62134 (n.s)
All Egyptian AMV isolates (* n* = 15)	13	0.971 (± 0.039)	0.03787 (± 0.01037)	0.05777	0.10479	0.533	-1.73774	-1.73401	-2.00010

*h* Number of Haplotypes, *Hd *Haplotype (gene) diversity, *π *nucleotide diversity, *dS* synonymous nucleotide diversity, *dN* nonsynonymous nucleotide diversity, *ω* *dN/dS *the evolutionary rate ratio which represents the average ratio between nonsynonymous and synonymous substitutions in sequence pairs (substitution rates), *n.s *not significant

The highest average number of nucleotide differences (k = 24.95238) was estimated for the GPI population (Supplemental S2). DNA divergence between GPI and GPII, GPIII and GPIV phylogroups was computed as described above. The results revealed that nucleotide diversity between the AMV phylogroups was (π (t)) 0.10090, 0.07996 and 0.09148 (Table 8). Pairwise Fst values showed strong genetic differentiation (Fst = 0.43709) between GPI and the other phylogroups (GPII, GPIII, and GPIV) of AMV populations (Table 9).

**Table 8:** DNA divergence between AMV populations using the computational method

**Variant group**	K	**π** (t)	Dxy	Da	**dN**	**dS**	**dN**/**dS** (**ω)**
**Egyptian isolates *Vs* isolates from global geographical origin**							
Asian	17.516	0.04597	0.05715	0.01459	0.05103	0.08388	0.594
Europe	22.103	0.05801	0.07477	0.03367	0.06410	0.11744	0.525
American	17.858	0.04687	0.06180	0.02817	0.05305	0.09540	0.539
Australian	15.675	0.04114	0.06404	0.04511	0.05678	0.09329	0.593
**GPI *Vs* phylogroups isolates**							
GPII	40.057	0.10090	0.11495	0.04640	0.00000	0.00000	0.00000
GPIII	30.066	0.07996	0.10652	0.04498	0.0000	0.00000	0.00000
GPIV	27.353	0.09148	0.10424	0.04166	0.00000	0.00000	0.00000
**Subpopupulation (delta) *vs* subpopulation (valley)**							
Delta/valley	43.152	0.11477	0.34339	0.02539	0.00000	0.00000	0.00000

*K* average number of nucleotide differences between sequences, *Dxy *Average number of nuc. subs. per site between populations, *Da *Number of net nucleotide substitutions per site between populations, dN, average number of nonsynonymous substitutions per nonsynonymous site; dS, average number of synonymous substitutions per synonymous site, with the Jukes and Cantor correction; dN/dS, (evolutionary rate ratio) average ratio between nonsynonymous and synonymous substitutions in sequence pairs

**Table 9:** Overall diversity, differentiation and gene flow estimates for AMV populations

AMV populations	Hd	πT	Ks*	Kst*	*P* value	Z*	*P* value	Snn	*P* value	Fst
Global geographic origins (*n* = 37)	0.99399	0.05559	2.48911	0.14277	0.0000 ***	4.94980	0.0000 ***	0.67568	0.0000***	0.26548
GPI, II,III, IV (*n* = 42)	0.98142	0.07528	1.90568	0.27595	0.0000 ***	4.79353	0.0000 ***	0.97619	0.0000***	0.43709
Delta, Valley (*n* = 15)	0.97143	0.11477	2.13245	0.22189	0.0060 **	3.41709	0.0110 *	0.86667	0.1550 n.s	0.07393

*Hd* Haplotype (gene) diversity, *Kst* Know sure thing, *Ks* Kolmogorov–Smirnov test, *n.s* not significant; *: 0.01 < *P* < 0.05; **: 0.001 < *P* < 0.01; ***: *P* < 0.001, Fst > 0.33 indicates infrequent gene flow; Fst < 0.33 suggests frequent gene flow

The nucleotide diversity in the AMV population in the CP gene region was examined to compare the genetic structure of the Egyptian AMV population with that of other AMV populations from around the world. Based on the analysis of the CP gene region, the AMV strains were distributed into five populations: Egypt, Australia, Asia (Iran, South Korea, and Saudi Arabia), Europe (France, Italy, New Zealand, Serbia. and England), and the American population, including North America (USA) and South America (Brazil, Mexico, and Argentina). At the worldwide geographical population level, the highest and lowest values of π and k were estimated for the Asian AMV within-population (π = 0.04787, k = 18.00) and American within-population (π = 0.02523, k = 7.722), respectively (Supplementary S2). The nucleotide diversity (π) level for the Egyptian AMV population comparing these subpopulations is depicted in Table 8 and Supplemental S2. The highest value of nucleotide diversity (π) was estimated for the European subpopulation (π = 0.05801, k = 22.103), and the lowest value of π was estimated for the Australian subpopulation (π = 0.04114, k = 15.675), as indicated in Table 8. The average number of nucleotide substitutions (Dxy) and the number of net nucleotide substitutions (Da) per site between populations were lowest in the Egyptian and Asian AMV populations (0.01459 and 0.05715, respectively) and highest (0.06404 and 0.04511, respectively) in the Egyptian and Australian AMV between populations (Table 8). The AMV populations according to the geographical global scale revealed significantly high Ks*, Z*, Snn and Kst* = 0.14277 values with a *P* value = 0.0000 *** and fixation index (*Fst* = 0.26548), respectively. Pairwise linkage between the worldwide AMV population from Asia, Europe, America, and Australia and the Egyptian AMV population revealed significantly high Ks*, Kst*, Z*, and Snn values (Table 9). Additionally, for each between-population, the dN values were lower than the dS values (dN/dS ratio < 1) (Table 8). The Asian isolate (dN/dS ratio (ω) = 0.594) and European isolate (ω = 0.525) populations had the greatest and lowest values, respectively (Table 8).

**Fig. 6:**
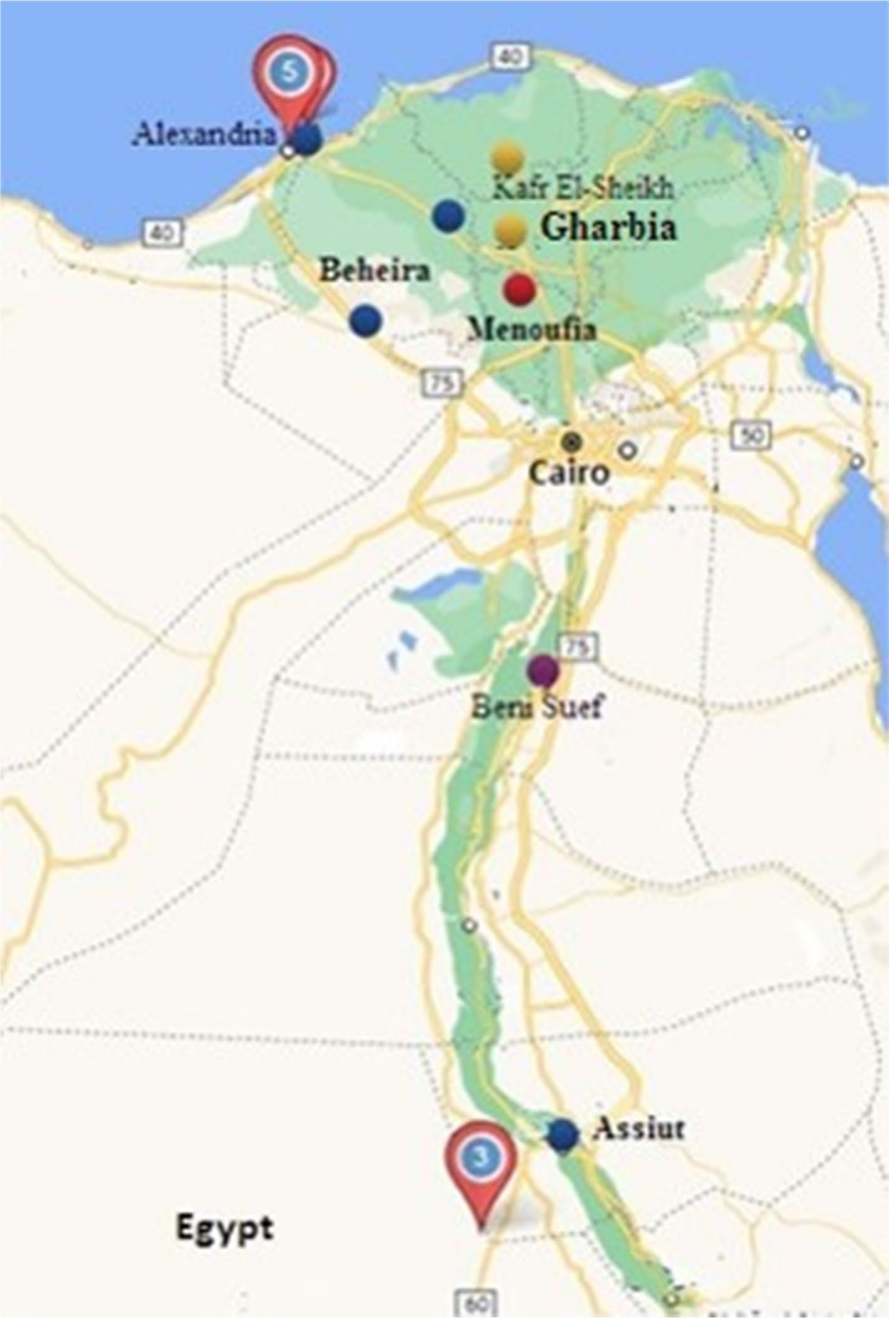
The Egyptian geographical locations of AMV isolates under investigation. The locations with the same color marker have isolates with similar sequence homology matrices. The map was generated using Maptive software according to the isolate location and its sequence homology matrix

To examine whether the Egyptian AMV population showed evidence of genetic diversity, the AMV isolates according to Egyptian geographic distribution were distributed in two subpopulations: the Nile delta (Delta) subpopulation (Shark, M123, EM143, EM99, AMV-EG, FER-AM1, E3, AMV77, and M3), including the present Egyptian isolates (AM1, AM2, AM3, and AM4), and the Nile River valley (Valley) subpopulation (AMV-Basil and Assiut). The distribution of the 15 AMV isolates, including the present isolates, according to their Egyptian geographic origin is shown in Fig. 6 and Tables 1 and 4. Thirteen different haplotypes were identified in the Egyptian AMV population (Table 7). The haplotype diversity (Hd) and nucleotide diversity (π) for the Valley subpopulation, including at least two AMV isolates (*n* = 2), were 1.0000 and 0.60372, respectively, and were higher than the diversity within the Delta subpopulation (*n* = 13) (0.96154 and 0.03229). Moreover, the Valley within-subpopulation genetic and nucleotide diversity was higher than the Delta and Valley between-subpopulation diversity together (0.97143 and 0.11477), with Kst* = 0.22189 significant *P* = 0.0060 **, a highly significant Z* = 3.41709 (*P* = 0.0110 *) and a Ks* = 2.13245 value. The level of genetic differentiation of Egyptian populations between Delta and Valley isolates was low, as revealed by *Fst* = 0.07393, implying complete genetic differentiation with frequent gene flow (Table 9). Tajima's D, Fu, and Li’s D and F statistical tests showed significant negative values for all 42 AMV isolates and all 15 Egyptian isolates (Table 7).

### Electron microscopy of purified AMV

**Fig. 7:**
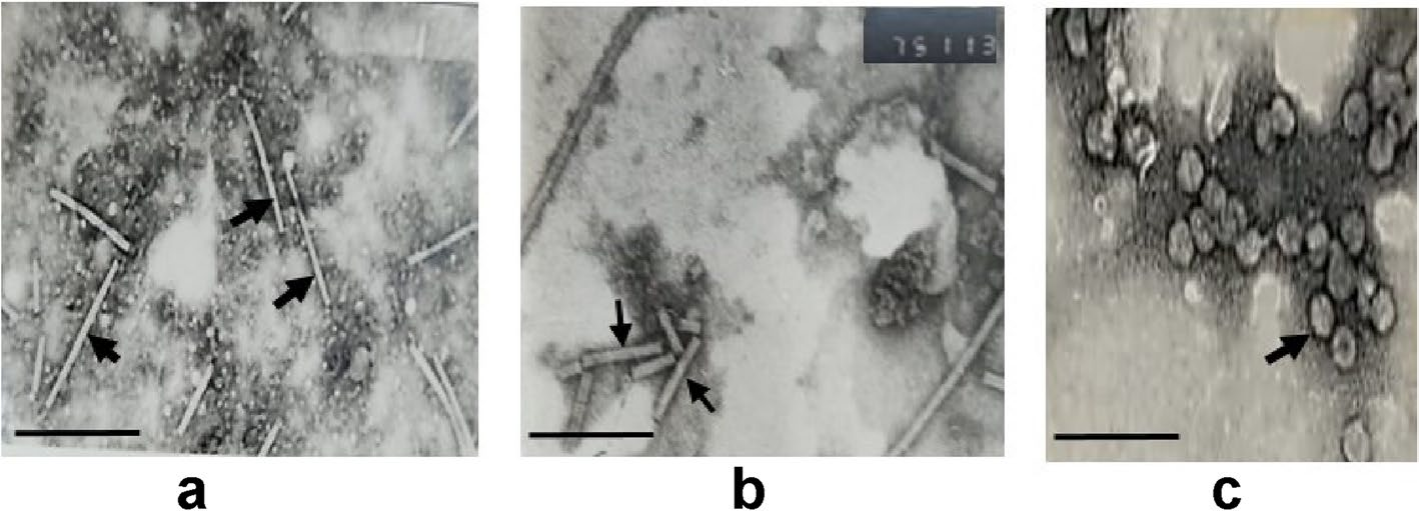
Electron micrographs of alfalfa mosaic virus particles that have been negatively stained. Electron micrograph of viral particles labelled with 2% uranyl acetate. **a** Virus particles having a characteristic bacilliform structure with long particles measuring 110 nm long are indicated by arrows. **b** The 68 nm long bacilliform particles are indicated by arrows. **c** 18 nm diameter spheroidal particles. The magnification is 150000x. The scale bar is 100 nm

The purified virus yielded approximately 26.82 mg/100 g of *N. glutimosa* fresh weight. Ultraviolet absorption studies of virus preparation from *N. glutimosa* revealed typical spectra of nucleoproteins with A max 260 and A min 242 nm. The A max/A min was 1.27, the A260/280 was 1.33 and the A280/260 was 0.075. Electron micrographs of the purified virus preparation showed spheroidal particles with a diameter of 18 nm (Fig. 7C) and three bacilliform particles with lengths of approximately 55, 68, and 110 nm (Fig. 7A and B). The majority of the longer particles were 110 nm in length (Fig. 7A).

## Discussion

In this study, we aimed to evaluate the genetic diversity and evolution of the resident population of AMV isolates in Egypt. To attain this purpose, forty-one samples of tomato, potato, alfalfa, and clover plants exhibiting virus–like symptoms (calico, bright yellow, and mosaic with slightly curved leaf margin symptoms) were collected and confirmed with AMV infection in different sites of Alexandria and Beheira governorates in Egypt by indirect ELISA and PCR. Eight out of 41 samples obtained were positive for AMV in indirect ELISA. Four samples were used as a source of the virus isolates in the present study and given the names AM1, AM2, AM3 and AM4 (collected from potato, tomato, alfalfa and clover host plants, respectively). AMV isolates were mechanically transmitted from naturally infected plants to healthy *N. glutinosa* seedlings and maintained in the greenhouse. Except for isolate AM2, all of the AMV isolates (AM1, AM3, and AM4) displayed the anticipated symptoms on the diagnostic hosts. The symptoms on *C. amaranticolor* and *N. glutinosa* plants matched those described by earlier studies for other strains of the virus [37–39]. The activity of antioxidant enzymes in response to each AMV isolate infection on *N. glutinosa* plants was assessed since host antiviral enzymes are thought to be one of the variables that affect the genetic diversity of plant viruses [40–43]. There were no significant differences in the antioxidant enzymatic activity of *N. glutinosa* plants infected with any AMV isolate, with the exception of the AM2 isolate, which showed a substantial variation in PPO enzyme activity. A comparison of the types of symptoms expressed by current AMV isolates on diagnostic host plants and the activity of host antiviral enzymes revealed that the natural host plants (potato, alfalfa, and clover) were all infected by the same AMV strains (AM1, AM3, and AM4, respectively), with the exception of tomato plants, which may have been infected by a different AMV strain (AM2). This result was similar to the findings of Fidan et al. [44]. RT‒PCR was used to detect and amplify the entire AMV-CP gene from total RNA extraction of *N. glutinosa* leaves mechanically inoculated with present AMV isolates. The size of the RT‒PCR amplicons (669 bp) was identical to that reported in previous studies [30, 45, 46]. Information about the level of diversity in AMV and comparison across AMV strains from other countries can help in building an appropriate strategy for detection and management of the virus [29, 30, 34, 35]. The SSCP pattern of RT‒PCR products of all AMV isolates under study was analysed and revealed a haplotype match between AM1, AM3 and AM4 isolates, while the AM2 isolate showed a different haplotype. SSCP patterns for all current AMV isolates indicated the presence of mixtures of AMV haplotypes with two predominant haplotypes. However, the SSCP pattern for the AM2 isolate resulted in only two dense bands, indicating the homogeneity of this isolate. Alignment of multiple sequences showed that the current AMV isolates had sequence identity of 98.2 to 99.5% at the nucleotide level between each other, which confirmed the haplotype frequency obtained by SSCP analysis. SSCP analysis is a powerful and accurate technique for estimating genetic diversity [47]. The CP gene sequence comparisons of four AMV isolates (AM1, AM2, AM3 and AM4) showed the highest nucleotide identity of 99.7% with the Gomchi isolate from South Korea infecting Gomchi (*Ligularia fischeri*) plants. Sequence comparison revealed that the degree of diversity across sixteen Egyptian AMV isolates, including the present isolates, ranged from 0.3 to 9.9%. The current data revealed the presence of different strains of AMV in Egypt. The Alexandria, Beheira, Kafr El-Sheikh, and Assiut isolates appear to be variants of the same AMV strain and showed a high degree of identity ranging between 95.1% and 99.7%, which indicates that most of the Egyptian AMV isolates are highly conserved among their strains. On the other hand, isolates (AMV-Basil and Menoufia) from Beni Suef and Menoufia were distant from all Egyptian isolates, as they have a nucleotide identity ranging from 90.1 to 93.8%, and thus they are considered to be fully distinct AMV strains. This reflects the diversity of the AMV population in Egypt and supports the hypothesis that there are two main strains of AMV [35]. Additionally, the percentage of nucleotide diversity of the present AMV sequences revealed a high degree of diversity (0.3—7.4%) among the 25 AMV isolates from thirteen geographical locations worldwide. These results support Parrella et al. [28], who demonstrated that variation in CP genes of AMV isolates worldwide was up to 7% at the nucleotide level. Previous investigations found that AMV isolates from various parts of the world had a significant level of diversity ranging from 4.0 to 6.1%, with one previous study reporting that the Egyptian strain, AMV-Basil, has as much as 8.7% variability [29, 36]. The CP gene of bromoviridae is typically highly varied, and CP is considered to be the most useful gene for building their phylogeny. The phylogenetic analysis of the entire viral coat protein gene has revealed that AMV isolates are grouped into four major groups (GP I, II, III and IV) and that most Egyptian isolates, including our current isolates, fall into a separate distinct group (GPI). The present AMV isolates and all previously reported Egyptian AMV isolates in the GPI group were most closely related to the Gomchi isolate from Korea, indicating that they may have originated from common ancestors. The GPI group containing Egyptian isolates was not present in all the group divisions mentioned by the authors [17, 28–32]. Our findings were consistent with those of Parrella et al*.* [28, 29], Milošević [31], Stanković et al. [32], and Engy *et al.* [34]. From our phylogenetic tree topology, we noticed that most isolates from Italy (126A, 195 AN, F 430), the United States (AMV 425 M and AMV 425 L) and France (Lye 80, Caa 1 and Dac 16) as well as isolates Fa.Fa. A, Ke.Ke. Pe and Ke.Ji. PO from Iran were individually grouped into subgroups. According to these results, we can assume that AMV is most likely distributed according to geographic distribution regardless of host type, as reported by Parrella et al*.* [17]. Our findings are consistent with previous reports [30, 32, 46] showing that AMV isolates could segregate into four or more groups. It assists in the diversification and adaptation of plant viruses to new hosts, both of which frequently lead to the formation of new variants and viral strains able to combat plant viral resistance [48]. The reassortment and recombination analysis could not detect any recombination signals in the CP gene sequences of the present AMV isolates (AM1, AM2, AM3, and AM4). On the other hand, among the Egyptian AMV isolates that retrieved from GenBank (old set), our study revealed four AMV isolates (AMV77, M3, AMV-Basil, and Menoufia) with potential reassortment/recombination events. The isolate EM99 was found to be the major donor (prevalent isolate) of recombination events. These data provide strong evolutionary evidence for the genetic diversity of AMV isolates by identifying potential recombination events involving parents from GPI and GPII lineages. The high degree of diversity and strain emergence found in this study (such as AMV-Basil and Menoufia) might be attributed to genome recombination and/or reassortment. This was confirmed by phylogeny, which revealed the presence of differently evolving strains that differed from the currently identified AMV group (GPI) and belonged to groups GPII and GPIII. The CP gene is one of the most widely used molecular markers to study genetic diversity and molecular evolution in different plant viruses [33]. The genetic diversity and molecular basis that controls AMV evolution were examined in this study using the CP gene of 42 AMV isolates. The present isolates and all other AMV strains employed in this investigation (*n* = 42) had a global selection pressure dN/dS (ω) = (0.6436), indicating that the AMV CP gene is under mild purifying negative selection (evolutionary constraint), as evidenced by the dN/dS (ω) ratio < 1, similar to what has been demonstrated for other plant viruses [49–51]. The nucleotide diversity of all Egyptian AMV strains (*n* = 15) was low (0.03787), indicating that the Egyptian AMV population was genetically stable with less variability, as reported by other authors [50]. However, Tajima's D, Fu, and Li's D* & F* statistic values were negative and significant for the entire Egyptian AMV population (*n* = 15, 11 from GenBank and 4 from this study), indicating that the predicted polymorphism was lower than expected. However, the negative nonsignificant Tajima's D and Fu and Li’s D* and F* values for the present Egyptian AMV population (*n* = 4) indicate that there are too many low-frequency polymorphisms. The genetic distinction of AMV populations was defined into three categories: phylogenetic groups, global geographical distribution and local geographic distribution. The level of genetic differentiation or the level of gene flow among AMV populations was measured by the fixation index (*Fst*) [52, 53]. AMV isolates were divided into four major phylogroup populations with an Fst value of 0.43709. Low nucleotide diversity and higher haplotype diversity suggested recent divergence in the AMV population under study. Further support for these results was found in highly significant values of Ks*, Kst*, Z*, Snn and (Fst > 0.33) statistics, which showed that the four phylogroup populations had very high genetic differentiation and infrequent gene flow (Fst = 0.43709), which is consistent with the phylogenetic analysis results. The DNA divergence between the populations of the GPI, GPII, GPIII, and GPIV phylogroups supported the results of the phylogenetic analysis and demonstrated that GPI is closely related to GPIII, with a lower nucleotide diversity value (π (t) = 0.07996). Pairwise lineage between the worldwide AMV geographic populations from Asia, Europe, America, and Australia and the Egyptian AMV population revealed significantly high Ks*, Kst*, Z*, and Snn values with an *Fst* value of 0.26548; this suggested complete genetic differentiation and frequent gene flow. The overall average diversity value for the global within-population diversity was lower than that for the between-population diversity, indicating population differentiation. This result confirmed that AMV populations differ geographically on a global scale with Kst* = 0.14277, *P* value = 0.0000 *** and fixation index *Fst* = 0.26548. The enumerating results for the evolutionary rate ratio *dN*/*dS* (ω) < 1 proposed purifying selection with restricting variability in the populations. The results of the phylogenetic analysis are supported by the lowest nucleotide diversity (0.04597), nucleotide average, and net number of nucleotide substitutions (Dxy and Da) values (0.05715 and 0.01459, respectively) in the pairwise lineage of AMV Egyptian and Asian between populations, which indicate that the Egyptian population is closely related to and shared evolutionary ancestors with the Asian AMV population. The negative significant Tajima's D, Fu and Li’s D and F values for all AMV isolates (*n* = 42) suggested that the AMV populations are expanding demographically. The level of genetic differentiation of Egyptian AMV populations according to the local geographical distribution between Delta strains and Valley strains was low (Fst = 0.07393), and Ks*, Kst*, and Z* were strongly supported by *P* values < 0.05, which implied complete genetic differentiation with frequent gene flow. The genetic diversity for the valley subpopulations was slightly higher than the diversity within the delta subpopulation, which became evident because the AMV-Basil isolate, which showed a low homology percentage (90.1%), was present in the valley subpopulations, whereas all haplotypes were present in the delta populations. The high frequency of AMV global distribution could be due to the wide host range of AMV and its transmission by various means, such as aphids, seeds and pollen. It has also been proposed that founder effects from plant material exchanged between various geographic regions may contribute to the genetic structure of AMV [51].

The deduced amino acid sequences of the tested AMV isolates that belong to group (I) revealed six nonconservative substitutions for amino acids at positions 29, 30, 34, 92, 117 and 175 for glutamine (Q^29^), serine (S^30^), threonine (T^34^), Valine (V^92^), Glutamic acid (E^117^) and Valine (V^175^), respectively. The amino acid Q^29^ and V^92^ substitutions were characterized for the Egyptian isolates reported in this study, while S^30^, T^34^, E^117^, and V^175^ substitutions were distinguished for group I (GPI) AMV isolates. Most of the amino acid substitutions for GP II and GP III occurred at previously reported positions [1, 17]. However, analysis of the complete genome sequence of Egyptian AMV could enable us to understand the evolutionary relationship of the virus more comprehensively.

AMV purification was performed to investigate whether these amino acid substitutions lead to the development of unusually long viral particles. The results showed that ultraviolet (UV) absorption of the purified virus preparation is typical for nucleoproteins. The yield of virus as well as its spectrophotometric data, i.e., A260/280 and A280/260, fall in the range reported for the corresponding data of AMV [37, 54, 55]. AMV is a multipartite virus that contains four particles of 18 nm diameter (three bacilliform and one spheroidal). The lengths of the bacilliform particles are between 55 and 68 nm, and the majority of longer particles are 110 nm in length. Therefore, such amino acid changes could be involved in the formation of unusually long virus particles, as reported by Thole et al*.* [3]. In Egypt, bacilliform virus-like particles with a length of approximately 130 nm were isolated from Assiut governorate [13]. Recently, typical bacilliform AMV particles with a length of 112.5 nm and a width of 57.5 nm were isolated from infected basil plants [36]. Our findings can be useful in investigations of aphid transmissibility and in silico epitope prediction of the AMV CP gene for GPI, particularly for the identification of immunologically relevant regions of Egyptian isolates. This study highlights the resident population of AMV isolates in Egypt and will help breeders who are working on different crops to breed varieties resistant to AMV. In addition, this study offers fundamental knowledge for creating a comprehensive disease control strategy against AMV in Egypt.

## Conclusions

This study provides the most comprehensive research on Egyptian AMV populations, focusing on the CP gene. Comparison of CP genes and phylogenetic analysis of Egyptian AMV isolates formed a distinct group (GPI) that was closely related to Gomchi isolate from South Korea. The study assessed antioxidant enzymes activity in *N. glutinosa* plants infected with each AMV isolate studied, showing that there was dissimilarity in the activity of the enzymes in only one AMV isolate (AM2). The study reveals strong evolutionary evidence for genetic diversity of AMV population, with potential recombination events in Egypt. This study highlights the genetic differentiation and evolution of the AMV population in Egypt. Moreover, it might help breeders working on different crops to select AMV-resistant crop varieties, and offer fundamental knowledge to develop a comprehensive disease-control strategy against AMV in Egypt.

## Methods

### Sources of virus isolates

The virus was isolated from naturally infected leaf samples of potato, tomato, alfalfa and clover plants. A total of forty-one samples (9, 11, 12, and 9 samples of each plant species) were collected after visual symptom inspection from three different locations in Alexandria and Beheira governorates during the 2019–2020 growing season (Table 1). Samples were tested with antiserum specific for AMV by indirect ELISA. Virus isolates were maintained in *Nicotiana glutinosa* plants in insect-proof greenhouse conditions for virus propagation and served as a source of the virus for subsequent studies. The relative incidence of the disease was calculated according to the following equation:

$$\mathrm{Percent}\;\mathrm{disease}\;\mathrm{incidence}\;(\mathrm{PDI})=(\mathrm{Number}\;\mathrm{of}\;\mathrm{symptomatic}\;\mathrm{plants}/\mathrm{Total}\;\mathrm{number}\;\mathrm{of}\;\mathrm{plants})\times100$$

### Reaction of diagnostic hosts

This study inoculated the following diagnostic hosts: *Chenopodium amaranticolor*, *Medicago sativa* L. (alfalfa), *Phaseolus vulgaris* cv. Contender, and *N. glutinosa*, which are known to produce symptoms characteristic of alfalfa mosaic virus, were used as previously described by [11, 37]. Virus inocula were prepared by grinding infected leaf tissue 1:10 (w/v) with a mortar and pestle in 0.1 M phosphate buffer, pH 7.0, containing 0.5% 2-mercaptoethanol. Leaves of plants to be inoculated were first dusted with carborundum (600 mesh) and then freshly inoculated with a prepared inoculum.

### Indirect ELISA

A total of 41 naturally infected samples of potato, tomato, alfalfa, and clover suspected of being infected with AMV were serologically tested with antiserum specific for AMV supplied by the Agricultural Genetic Engineering Research Institute (AGERI), ARC, Egypt. Indirect ELISA was performed using antigen-coated plates as described by Koenig [56] and modified by Fegla et al*.* [57]. Extracts from infected and healthy potato, tomato, alfalfa, and clover plants were diluted using a coating buffer (0.05 M carbonate, pH 9.6) to 1:10. The ELISA values were measured by a Multi Skan Ex ELISA reader at a wavelength of 405 nm. The absorbance reading values of at least twice that of the healthy control were considered positive.

### Antioxidant enzyme assay

To prepare enzyme extracts, one gram of *N. glutinosa* leaf tissue inoculated with the current AMV isolates (AM1, AM2, AM3, and AM4) representing one ELISA-positive plant for each host (samples with asterisk value in Table 2) was immersed in liquid nitrogen and pulverized into a powder. The tissue powder was homogenized with 4 ml of 0.1 M phosphate buffer (pH 7.0) containing 0.1 M Na-EDTA and 1% (w/v) polyvinyl pyrrolidone (PVP). Filtration of the extracts was accomplished with a nylon cloth. After that, the extracts were centrifuged at 10,000 × g for 20 min at 4 °C, and the supernatant was collected and stored at -80 °C for further analysis of enzyme activity [58]. Superoxide dismutase (SOD) activity was assayed by measuring its ability to inhibit the photochemical reduction of nitro blue tetrazolium (NBT) chloride [59]. The disappearance rate of H_2_O_2_ at 240 nm was used to determine the activity of catalase (CAT) [60]. The ascorbate peroxidase (APX) activity was assayed according to a previously described method [61]. The initial rate of quinone production, as indicated by an increase in absorbance units (AUs) at 240 nm, was used to measure polyphenol oxidase (PPO) activity. An increase in absorbance of 0.001 min^−1^ was taken as a unit of enzyme activity [62]. Data were statistically analysed using Costate Statistics Software (version 7.7 beta). The level of significance was determined by L.S.D. comparisons at the *p* = 0.05 probability level.

### Assay of hydrogen peroxide (H_2_O_2_) generation

The hydrogen peroxide measurement method was previously described [63].

### RNA extraction and RT‒PCR

Four samples with severe AMV mosaic symptoms and high ELISA values, one for each host, were selected for further molecular analysis (the selected samples are with asterisk ELISA reading values in Table 2). Total RNA was extracted from infected tissues using a Total RNA Mini Kit for plants (Geneaid) according to the manufacturing manual/guide protocol. The isolated RNA was used as a template for the one-step RT‒PCR. One-step RT‒PCR was performed using the Verso™ one-step RT‒PCR kit (Thermo Scientific) according to the instruction manual. The reaction was performed in a total volume of 25 μl of the amplification mixture using 12 ng of RNA and 20 μM of each CP gene-specific primer [64], AMV-F2 forward primer (5’-ATCATGAGTTCTTCACAAAAGAA-3’) and AMV-R2 reverse primer (5’-TCAATGACGATCAAGATCGTC-3’), 12.5 µL of 2 × One-Step RT Master Mix, 1.25 µL of RT-enzyme enhancer and 0.5 µL of verso enzyme mix. The primer set was designed by Xu and Nie [30] to amplify the full-length coat protein (CP) gene (669 bp) of AMV RNA3. RT‒PCR amplification was carried out using a thermocycler (Uno), starting with a reverse transcription reaction at 50 °C for 15 min and 95 °C for 5 min, followed by 35 cycles for PCR amplification at 94 °C for 30 s, 58 °C for 30 s and 72 °C for 30 s, with a final extension step at 72 °C for 7 min. RT‒PCR products were analysed directly with 1% prestained agarose gel using EZView stain and gel electrophoresis [65].

### SSCP Analysis

Single-strand conformational polymorphism (SSCP) analysis was performed as previously described [66, 67]. One microliter of the RT‒PCR product was added to 9 μl of denaturing solution (95% formamide, 20 mM EDTA (pH 8.0), 0.1% bromophenol blue) and boiled for 5 min followed by cooling on ice. Samples were resolved in 8% polyacrylamide gels at 200 V and 4 °C for 3 h.

### Sequencing and phylogenetic analysis

RT‒PCR products showing identical or different SSCP patterns were purified using a PCR purification kit (Geneaid) according to the manufacturer’s protocol. The purified RT‒PCR products were ligated into the pCR^TM^2.1-TOPO™ cloning vector (Invitrogen, Carlsbad, CA, USA) according to the manufacturer’s instruction manual. The clones (one clone for each isolate) were submitted for bidirectional DNA sequencing by an analysis company in Egypt using a Perkin Elmer ABI Prism 377 DNA Sequencer. The full-length coat protein nucleotide sequences were analysed using DNAMAN 8.0 software (Lynnon Biosoft, Quebec, Canada). One set of new nucleotide sequence data of Egyptian AMV isolates (the current AMV isolates under this study) and one old set of Egyptian AMV strains retrieved from GenBank and infecting different crops from different locations previously reported in Egypt (12 isolates) were compared with other international AMV isolates available in GenBank. The geographic origin, accession numbers, and natural hosts of the 38 reference isolates used in nucleotide sequence comparisons and phylogenetic analysis are shown in Table 4. Multiple sequence alignment along with the sequences of 38 reference strains from GenBank was performed using QIAGEN CLC Genomics Workbench 22.0.2 software (https://digitalinsights.qiagen.com). Amino acids (a.a.) were translated using the ExPASy Translate tool (http://us.ExPASy.org/tools/dna.html). The QIAGEN CLC Genomics Workbench 22.0.2 software (QIAGEN) was used to generate the circular cladogram of the neighbor-joining (NJ) phylogenetic tree with 1000 bootstrap replicates. Alignment of deduced amino acids was performed using DNAMAN 8.0 software.

### Recombination and genetic diversity analysis

The RDP v.4.16 program with its implemented algorithms [RDP (R), GENECONV (G), Chimaera (C), MaxChi (M), BOOTSCAN (B), SISCAN and 3 Seq (T)] and a statistically corrected *P* value cut-off of 0.05 was used to identify likely occurrences of reassortment/recombination in Egyptian AMV isolates. The rates of synonymous (dS) and nonsynonymous (dN) substitutions among protein-coding sequences were compared to determine the nucleotide diversity (π) value and the degree of selective pressure using DnaSP6 v6.12.03 software [52]. The evolutionary rate ratio (dN/dS) is frequently used to identify protein sites that undergo purifying selection (dN/dS < 1), evolve neutrally (dN/dS ≈ 1), or undergo positive, diversifying selection (dN/dS > 1) [49]. This metric measures how quickly amino acids change in protein constituents relative to synonymous changes in AMV populations. CP gene sequences from 42 AMV isolates (four from this study and 38 AMV isolates retrieved from GenBank) (Table 4) were used to analyse population divergence, estimate genetic diversity parameters and determine phylogenetic relatedness. They were also used to examine the key evolutionary factors that have shaped the genetic structure of AMV globally. Using DnaSP version 6.12.03 software [52], population genetic parameters were computed based on variant groups and regional distributions. The hypothesis of neutral selection operating on the CP gene was assessed using statistical tests implemented in DnaSP6, such as Tajima's D [68]. Several statistics, including Ks*, Kst*, Z*, and Snn based on permutation statistical tests with 1,000 replicates, were used to investigate genetic divergence between populations [69]. If the statistical tests (Ks*, Kst*, Z*, and Snn) substantially support the null hypothesis of no genetic differentiation and have *P* values < 0.05, then the null hypothesis is rejected. The fixation index (Fst) was used to determine how genetically distinct or how much gene flow occurred across AMV populations [53]. Fst can range in value from 0 (full gene flow and no genetic differentiation) to 1 (null gene flow and complete genetic differentiation). Fst > 0.33 typically suggests infrequent gene flow, while Fst < 0.33 generally indicates frequent gene flow [52].

### Purification of AMV

AMV was purified as per the protocol described [11]. One hundred grams of *Nioctiana glutinosa* plants systemically infected with AMV were collected at 20–25 dpi and homogenized (1 g/1 ml) for 5 min in a blender with freshly prepared 0.1 M phosphate buffer (pH 7.5) containing 1% 2-mercaptoethanol. The extract was passed through a double layer of cheesecloth and stirred vigorously for 10 min with an equal volume of a 1:1 cold mixture of n-butanol and chloroform. The emulsion was broken by low-speed centrifugation at 8000 rpm (7.741 xg) at 4 °C using a Beckman centrifuge Model J. TB-024F with a JS-13–1 rotor. The clarified extract was left at 4 °C for 16 h and then centrifuged at 8000 rpm for 10 min. The virus was concentrated by two cycles of differential centrifugation. The first cycle included centrifugation at 35,000 rpm (33.868 xg) for 120 min in a Beckman L7-65 ultracentrifuge (Rotor type 70–1 T1). The resulting pellets were resuspended in 0.01 M phosphate buffer, pH 7.5, containing 0.001 M ethylene diamine-tetra acetic acid (EDTA)-(suspending buffer), for 60 min before receiving a low speed centrifugation at 6000 rpm (5.806 xg) for 10 min. The volume of the suspending buffer was ${}^{1}\!\left/ \!{}_{5}\right.$ of the original clarified extract. A second similar cycle of differential centrifugation was performed. Finally, the resulting pellet was suspended in 2 ml of the suspended buffer. A 260/280, A 280/260, and A max/A min as well as virus concentration were estimated. Virus concentration was calculated by assuming an extinction coefficient (E260 nm 0.1%) of 5.2 [40].

### Electron microscopy (EM) of purified AMV

Formavar-coated nickel grids were floated on drops (20 µl) of purified virus preparation for 5 min. After rinsing with distilled water, the grids were stained with 2% uranyl acetate and examined with a Jeol JEM-1400 TEM electron microscope, Faculty of Science—Alexandria University.

### Supplementary Information

**Additional file 1.** The Word file contains a full-length agarose gel electrophoresis image of RT‒PCR products of the CP gene of AMV (displayed in Fig. 3) and a full-length SSCP blot of the CP gene amplicon (displayed in Fig. 4 in this study). The SSCP pattern of the current AMV isolates is marked in the blot.

**Additional file 2.** Text file contains the protein identity matrix for AMV isolates used in this study.

**Additional file 3: Supplement S1.** Nucleotide identity matrix among the present AMV isolates (AM1, AM2, AM3 and AM4) and reported 12 AMV coat protein nucleotide sequences of Egyptian isolates. **Supplement S2.** AMV within-populations genetics parameters for the AMV CP gene sequences.

## Data Availability

All data generated or analysed during this study are included in this published article [and its supplementary information files]1. The datasets supporting the conclusions of this article are included within the article and its Additional files 1 and 2. 2.The nucleotide sequences for the CP gene of the present AMV isolates in this investigation have been deposited in the GenBank database under the following accession numbers: OM105666 to OM105669.
