# Supplementary material for: Molecular characterization and evolution of the resident population of some alfalfa mosaic virus (AMV) isolates in Egypt
Source: BMC Microbiol. 2023 Sep 18;23:261. doi: 10.1186/s12866-023-03003-8 (PMC10506327; doi:10.1186/s12866-023-03003-8)
Supplement: Supplementary file 1 — Additional file 1. The Word file contains a full-length agarose gel electrophoresis image of RT‒PCR products of the CP gene of AMV (displayed in Fig. 3) and a full-length SSCP blot of the CP gene amplicon (displayed in Fig. 4 in this study). The SSCP pattern of the current AMV isolates is marked in the blot. [file 12866_2023_3003_MOESM1_ESM.docx]

**Paper Title**: Molecular Characterization and Evolution of the Resident Population of Some Alfalfa mosaic virus (AMV) Isolates in Egypt


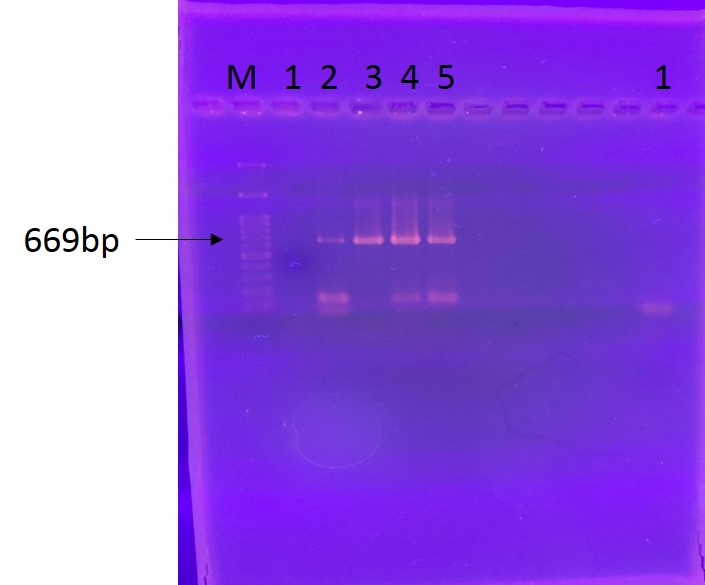


**Fig. 3** 1% agarose gel electrophoresis analysis of AMV CP gene amplicons obtained by RT-PCR from mechanically inoculated *N. glutinosa* leaves. M: 100 bp DNA Ladder (GeneDireX, Inc.); lane 1: uninfected healthy *N. glutinosa* plant; lane 2: isolate AM1; Lane 3: isolate AM2; Lane 4: isolate AM3; and Lane 5: isolate AM4. 'Full-length gel is presented in Additional File 1', Figure 3.


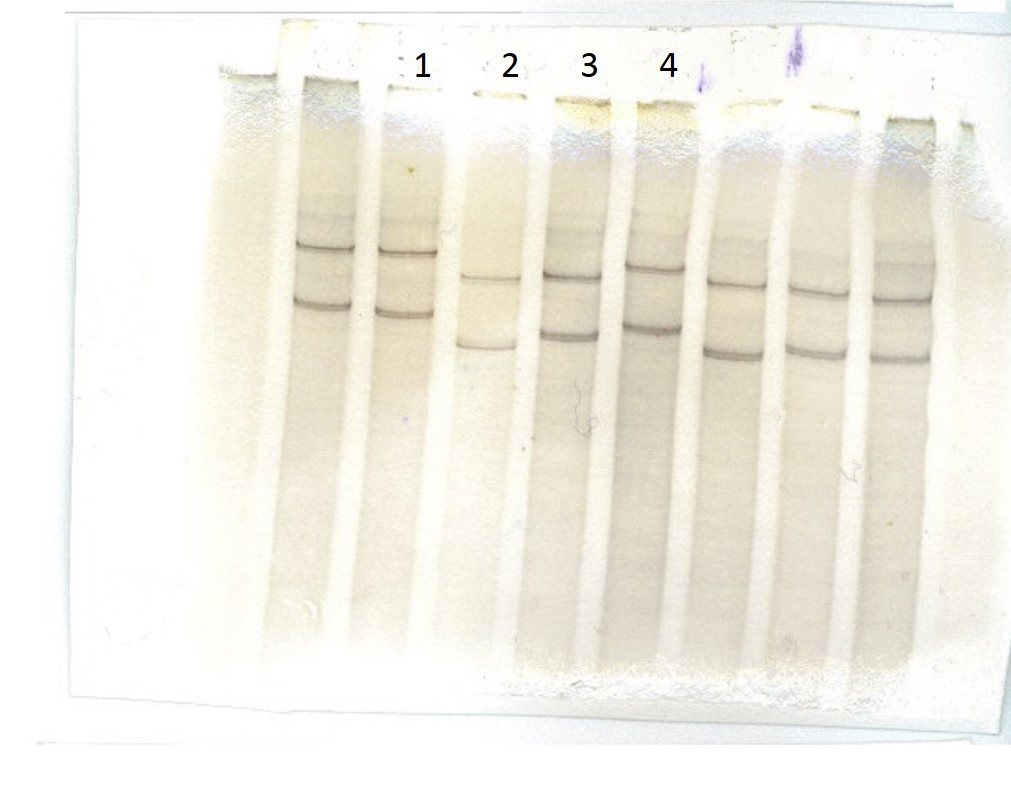


**Fig. 4.** SSCP patterns of RT-PCR products of all tested AMV isolates; lane **1:** AM1 isolate; Lane **2:** AM2 isolate; Lane **3:** AM3 isolate and Lane **4:** AM4 isolate.
