# Supplementary material for: Molecular characterization and evolution of the resident population of some alfalfa mosaic virus (AMV) isolates in Egypt
Source: BMC Microbiol. 2023 Sep 18;23:261. doi: 10.1186/s12866-023-03003-8 (PMC10506327; doi:10.1186/s12866-023-03003-8)
Supplement: Supplementary file 3 — Additional file 3: Supplement S1. Nucleotide identity matrix among the present AMV isolates (AM1, AM2, AM3 and AM4) and reported 12 AMV coat protein nucleotide sequences of Egyptian isolates. Supplement S2. AMV within-populations genetics parameters for the AMV CP gene sequences. [file 12866_2023_3003_MOESM3_ESM.docx]

**Paper Title**: Molecular Characterization and Evolution of the Resident Population of Some Alfalfa mosaic virus (AMV) Isolates in Egypt

Supplement **S1**: Nucleotide identity matrix among the present AMV isolates (AM1, AM2, AM3 and AM4) and reported 12 AMV coat protein nucleotide sequences of Egyptian isolates.


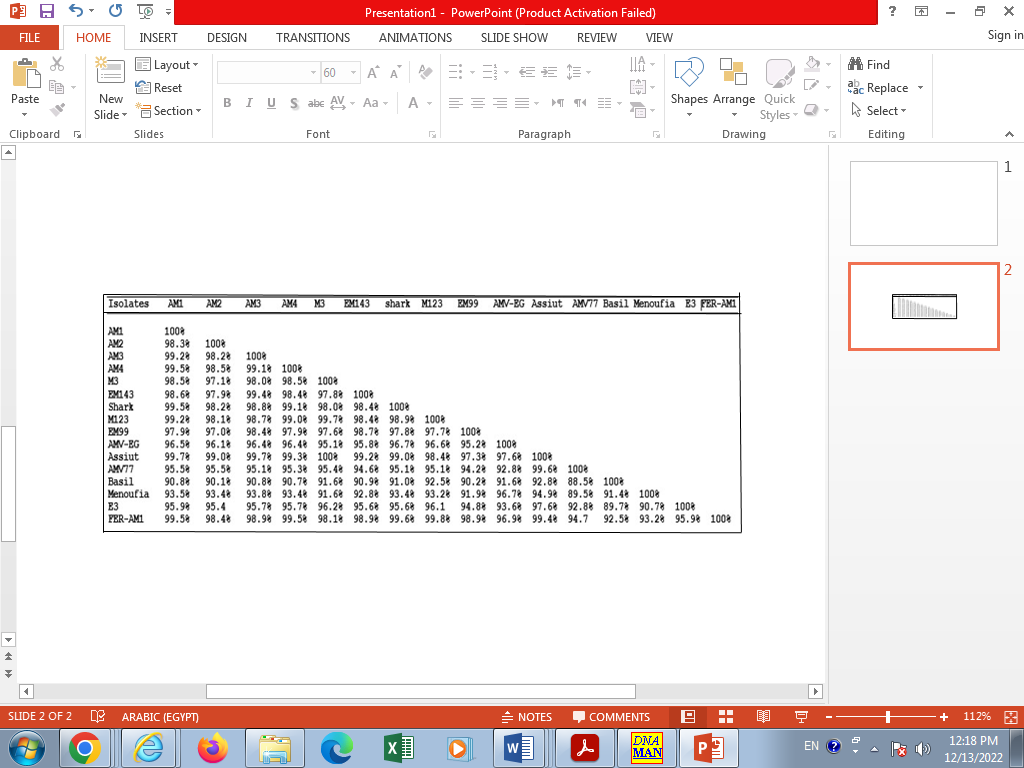


Supplement **S2**: AMV within-populations genetics parameters for the AMV CP gene sequences

| **Variant group** | **S** | **h** | **Hd** | **K** | **π** |
| --- | --- | --- | --- | --- | --- |
| **PHYLOGROUP population** | | | | | |
| GPI | 170 | 10 | 0.85714 | 24.95238 | 0.08976 |
| GPII | 25 | 6 | 1.00000 | 11.00000 | 0.03957 |
| GPIII | 32 | 18 | 0.99415 | 6.67836 | 0.02402 |
| GPIV | 11 | 2 | 1.00000 | 11.00000 | 0.03957 |
| **Egyptian and local geographic subpopulations** | | | | | |
| Egyptian (n=15) isolate | 74 | 13 | 0.97143 | 14.42857 | 0.03837  (±0.01037) |
| Current isolates (n=4) | 24 | 4 | 1.000 | 12.33 | 0.01855  0.00667)) |
| Delta (n=13)  Sub-popul. 1 | 59 | 11 | 0.96154 | 12.14103 | 0.03229 |
| Valley (n=2)  Sub-popul. 2 | 227 | 2 | 1.00000 | 227.0 | 0.60372 |
| **World wide Geographic populations** | | | | | |
| Asian population | 37 | 5 | 1.00000 | 18.00000 | 0.04787 |
| European population | 51 | 12 | 1.00000 | 16.89394 | 0.04493 |
| Australian population | 26 | 4 | 0.90000 | 11.20000 | 0.02979 |
| American population | 39 | 5 | 1.000 | 7.722 | 0.02523 |
|  |  |  |  |  |  |

^S: S, number of polymorphic (segregating) sites; K, average number of nucleotide differences between sequences; π, nucleotide diversity, Delta: isolates and strains collected from Nile river delta governorates used in this study (Alexandria , Menoufia, Kafr El-Sheikh, Beheira and Gharbia), Valley: strains from Nile river valley governorates used in this study (Beni- Suef and Assiut).^
